# *Necator americanus Ancylostoma* Secreted Protein-2 (*Na*-ASP-2) Binds an Ascaroside (ascr#3) in Its Fatty Acid Binding Site

**DOI:** 10.3389/fchem.2020.608296

**Published:** 2020-12-17

**Authors:** Ola El Atab, Rabih Darwiche, Nathanyal J. Truax, Roger Schneiter, Kenneth G. Hull, Daniel Romo, Oluwatoyin A. Asojo

**Affiliations:** ^1^Division of Biochemistry, Department of Biology, University of Fribourg, Fribourg, Switzerland; ^2^Department of Biological Chemistry and Molecular Pharmacology, Harvard Medical School, Boston, MA, United States; ^3^Department of Chemistry and Biochemistry & The CPRIT Synthesis and Drug-Lead Discovery Laboratory, Baylor University, Waco, TX, United States; ^4^Department of Chemistry and Biochemistry, Hampton University, Hampton, VA, United States; ^5^National School of Tropical Medicine, Baylor College of Medicine, Houston, TX, United States

**Keywords:** venom allergen-like (VAL), TAPs [testis specific proteins (Tpx)/antigen 5 (Ag5)/pathogenesis related-1 (PR-1)/Sc7], CAP [cysteine-rich secretory protein (CRISP)/antigen 5/pathogenesis related-1 (PR-1)], lipid binding, sperm coating protein (SCP)

## Abstract

During their infective stages, hookworms release excretory-secretory (E-S) products, small molecules, and proteins to help evade and suppress the host's immune system. Small molecules found in E-S products of mammalian hookworms include nematode derived metabolites like ascarosides, which are composed of the sugar ascarylose linked to a fatty acid side chain. The most abundant proteins found in hookworm E-S products are members of the protein family known as *Ancylostoma* secreted protein (ASP). In this study, two ascarosides and their fatty acid moieties were synthesized and tested for *in vitro* binding to *Na*-ASP-2 using both a ligand competition assay and microscale thermophoresis. The fatty acid moieties of both ascarosides tested and ascr#3, an ascaroside found in rat hookworm E-S products, bind to *Na-ASP-2's* palmitate binding cavity. These molecules were confirmed to bind to the palmitate but not the sterol binding sites. An ascaroside, oscr#10, which is not found in hookworm E-S products, does not bind to *Na*-ASP-2. More studies are required to determine the structural basis of ascarosides binding by *Na*-ASP-2 and to understand the physiological significance of these observations.

## Introduction

*Necator americanus* and *Ancylostoma duodenale* are hookworms causing a disease burden of over 22 million disability-adjusted life years (de Silva et al., 2003; Hotez, 2007; Murray et al., 2013; Diemert et al., 2018). The most abundant proteins secreted by third-stage infective larvae (L3) of *N. americanus* upon host entry are *N. americanus Ancylostoma* secreted protein 1 (*Na*-ASP-1) and *N. americanus Ancylostoma* secreted protein 2 (*Na*-ASP-2) (Hotez et al., 2003). These *Ancylostoma* secreted proteins are the major protein components of the L3 excretory-secretory (E-S) products that facilitate the evasion and suppression of the host's immune system and have been found in parasitic nematodes (Hawdon et al., 1995, 1996, 1999; Hawdon and Hotez, 1996; Gao et al., 2001; Zhan et al., 2003; Asojo et al., 2018; Darwiche et al., 2018). ASPs belong to the SCP/TAPS (sperm-coating protein/Tpx/antigen 5/pathogenesis related-1/Sc7) superfamily of proteins, NCBI domain cd00168 or Pfam PF00188 (Gibbs et al., 2008). Members of the SCP/TAPS superfamily are also implicated in other biological phenomena, including cellular defense such as plant responses to pathogens, sexual reproduction, and human brain tumor growth (Hawdon et al., 1999; Ding et al., 2000; Gao et al., 2001; Zhan et al., 2003; Gibbs et al., 2008, 2010).

SCP/TAPS proteins have either one or two ~15 kDa cysteine-rich CAP domains (cysteine-rich secretory protein, antigen 5, and pathogenesis-related 1) as typified by the structures of *Na*-ASP-2 (one CAP domain) and *Na*-ASP-1 (two covalently linked CAP domains). The CAP domain has multiple cavities and verified ligand-binding regions, and the first to be identified was a large central cavity that may contain a tetrad of residues, two His and two Glu that bind divalent cations including Zn^2+^ and Mg^2+^(Gibbs et al., 2008; Wang et al., 2010; Asojo et al., 2011, 2018; Mason et al., 2014; Darwiche et al., 2018). Distinct lipid-binding sites verified in SCP/TAPS proteins include a caveolin-binding motif (CBM) of the yeast CAP proteins required for *in vivo* transport of cholesterol and a hydrophobic channel formed by conserved central helices that bind fatty acids (Xu et al., 2012; Kelleher et al., 2014; Darwiche et al., 2016, 2018; Asojo et al., 2018). Sterols and fatty acid bind at these two different and independent binding sites on the CAP domain of SCP/TAPS proteins have been confirmed for multiple CAP proteins and with mutagenesis studies (Darwiche and Schneiter, 2017; Darwiche et al., 2017a; Asojo et al., 2018).

We previously reported the crystal structure of *Na*-ASP-2 and its sterol binding and transport properties (Asojo et al., 2005; Darwiche et al., 2018). The impetus for this current study is to investigate the fatty acid binding properties of *Na*-ASP-2. We also investigate if the major small molecules in E-S products (ascarosides) bind to *Na*-ASP-2, a major protein in the E-S. This is of interest because ascarosides are composed of the sugar ascarylose linked to a fatty acid moiety. Furthermore, we investigated the binding of two structurally similar ascarosides ascr#3 and oscr#10 and their fatty acid moieties. We chose these ascarosides because a high relative abundance of ascr#3 was detected in E-S products from both the infective juvenile and adult stages of rat hookworm (*Nippostrongylus brasilensis*) by HPLC-MS whereas oscr#10 was not present (Choe et al., 2012). *N. brasilensis* is a murine model of human hookworm infection and has a similar E-S proteins expression profile as the major human hookworm *N. americanus* (Camberis et al., 2003; Sotillo et al., 2014). We present the results of the binding studies as well as methods for the efficient synthesis of both ascarosides and their fatty acid moieties.

## Materials and Methods

### Synthesis of Ascarosides and Fatty Acids

Details about the synthesis of the ascarosides and fatty acids moieties produced for our studies are described in supplementary methods. Briefly, we synthesized the intact fatty acid side moieties and coupled them directly to a protected ascarylose followed by final deprotections.

### Expression and Purification of Pry1 and *Na*-ASP-2

Recombinant proteins were produced using both *P. pastoris* for untagged protein and *E. coli* for hexa-histidine tagged protein. Untagged protein was produced as previously reported (Asojo et al., 2005; Darwiche et al., 2016). DNA encoding for Pry1 and *Na*-ASP-2 were amplified by PCR and cloned into *NcoI* and *XhoI* restriction sites of pET22b vector (Novagen, Merck, Darmstadt, Germany), which contains a pelB signal sequence to direct the secretion of expressed protein into the periplasmic space. Plasmids were transformed into *Escherichia coli* BL21 and proteins were expressed with a C-terminal polyhistidine-tag. Protein expression was induced overnight with lactose at 24°C. Cells were collected, lysed and incubated with nickel-nitrilotriacetic acid beads (Ni^2+^-NTA) as per the manufacturer instructions (Qiagen, Hilden, Germany). Beads were washed and proteins were eluted in 60 mM NaH_2_PO_4_, 300 mM NaCl and 300 mM imidazole, pH 8.0. Prior to microscale thermophoresis experiments, proteins were applied to Zeba^TM^ spin desalting columns (Thermo scientific) and the buffer was exchanged to 60 mM NaH_2_PO_4_, 300 mM NaCl, pH 8.0.

### *In vitro* Radioligand Lipid Binding Assay

The radioligand binding assay was performed as described previously (Im et al., 2005; Choudhary and Schneiter, 2012). Hundred pmol of purified untagged CAP protein (*Na*-ASP-2 or Pry1) in binding buffer (20 mM Tris, pH 7.5, 30 mM NaCl, 0.05% Triton X-100) was incubated for 1 h at 30 °C with different concentrations of either [^3^H]-cholesterol or [^3^H]-palmitic acid. Protein was removed from unbound ligand by adsorption to Q-sepharose beads (GE Healthcare, USA), the beads were washed, protein was eluted and the protein-bound radioligand was quantified by scintillation counting. For competition binding assays, specified concentrations of unlabeled cholesterol, palmitic acid or ligands, were included in the binding reaction. Non-specific binding was determined by performing the assays without the addition of protein. Statistical significance of data was analyzed by multiple *t*-test (GraphPad Prism, La Jolla, CA).

### Microscale Thermophoresis

Microscale thermophoresis was performed using a Monolith NT.115 from Nanotemper Technologies (Munich, Germany) (Seidel et al., 2012; Shang et al., 2012; Zillner et al., 2012). His-tagged protein (Pry1 or *Na*-ASP-2) was fluorescently labeled using the RED-tris-NTA His-tag protein labeling kit (Nanotemper Technologies). Labeled protein (Pry1 or *Na*-ASP-2) was subsequently added to serial dilution of unlabeled ligand (ascarosides or their fatty acid moieties) in binding buffer (20 mM Tris pH 7.5, 30 mM NaCl, 0.05% Triton X-100). Each sample was loaded into standard glass capillaries, and measurements were performed at 60% laser power setting. Dissociation constant *K*_*d*_ was obtained by plotting the normalized fluorescence (Fnorm) against the logarithm of ligand concentration. Experiments were performed in triplicates and the *K*_*d*_ model of the MO Affinity Analysis software (Nanotemper Technologies, Munich, Germany) was used for data fitting.

## Results

### Synthesis of Ascarosides and Fatty Acids

**Figure 1:**
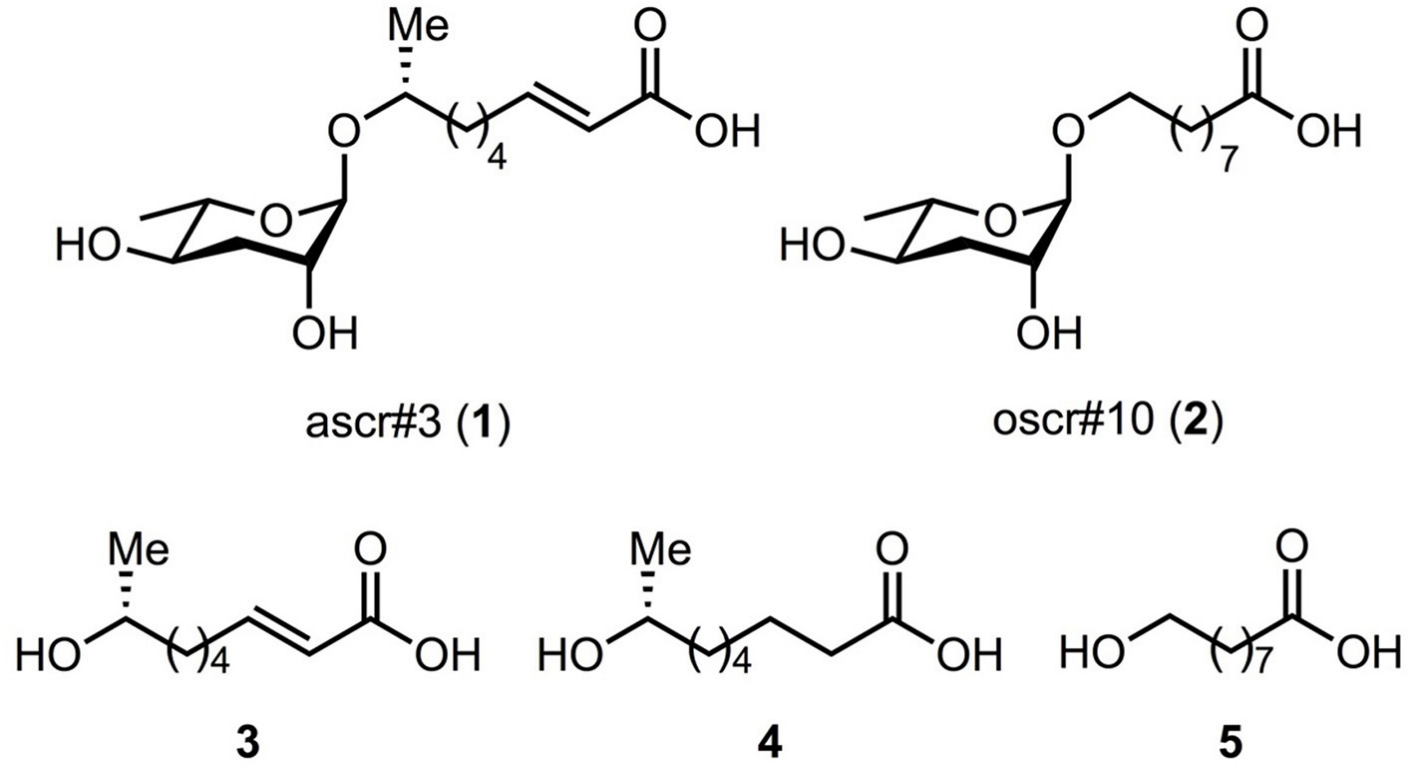
Targeted ascarosides and their fatty acid moieties. The corresponding ascarosides are ascr#3 (**1**); oscr#10 (**2**) and their side chain moieties are **3-5**. Compound names are **3** = (*R*)-8-hydroxynonanoic acid, **4** = (*R, E*)-8-hydroxynon-2-enoic acid, and **5** = 9-hydroxynonanoic acid.

**Figure 2:**
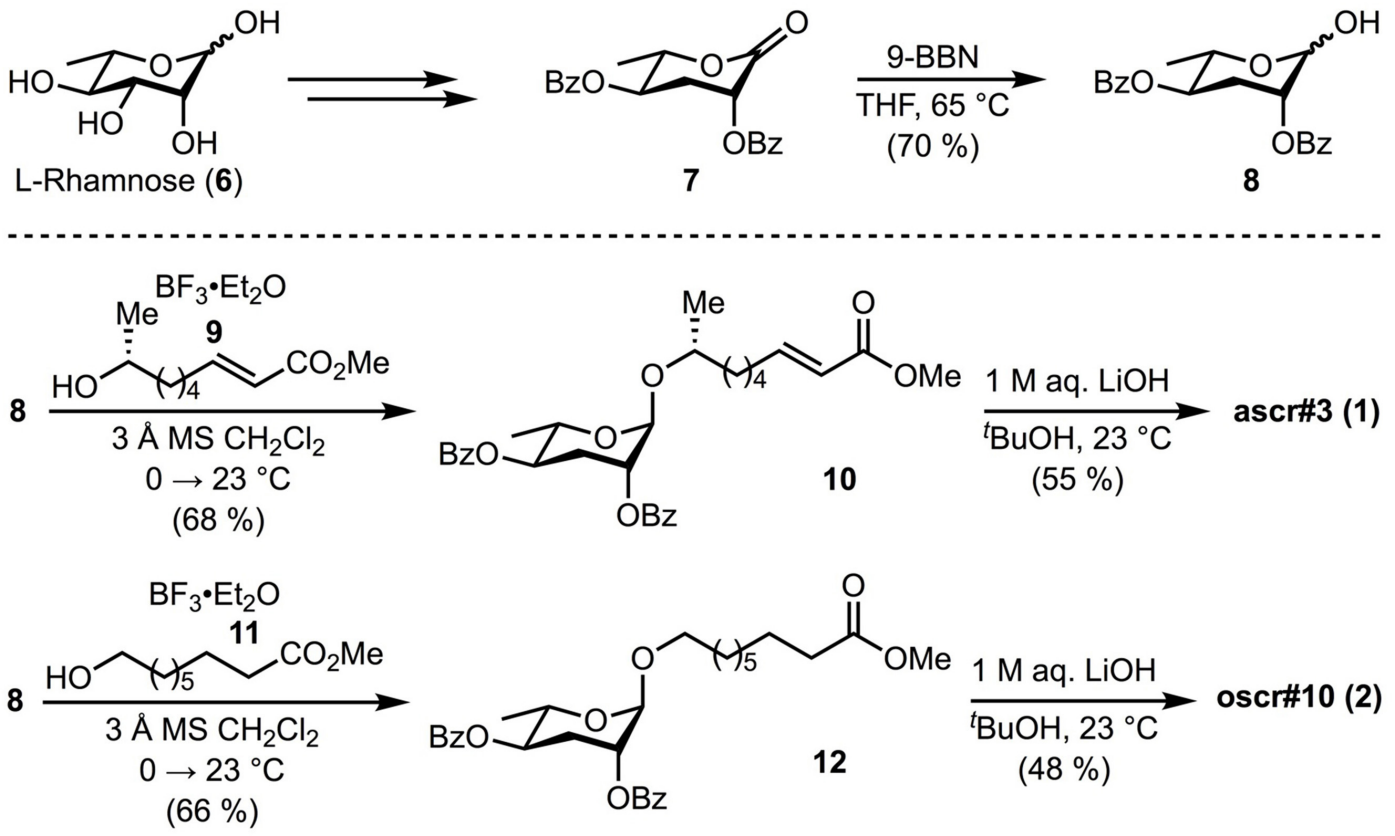
Synthesis of ascarosides. The synthetic pathway designed for protected ascarylose **8**, ascr#3 (**1**), oscr#10 (**2**) are illustrated. Detailed synthesis methods are described in the supplementary methods.

Since the ascarosides and fatty-acid moieties (Figure 1) required for our studies were not readily available, we adapted existing methods for their synthesis. Benzoyl protected ascarylose **8** was prepared as previously reported by Jeong et al. from commercially available *L*-rhamnose **6** (Jeong et al., 2005) with the exception of a modified final reduction. The previously reported reduction of lactone **7** with disiamyl borane (Jeong et al., 2005) proved irreproducible in our hands, resulting in incomplete conversion and low overall yields (~40%). Thus, an alternative was identified involving reduction with 9-BBN to provide the desired lactol **7** in improved yield (70%). With protected ascarylose **8** in hand, we next studied glycosylation at C1 to append the fatty acid side chain present in the targeted ascarosides. Previous synthetic strategies to these targets involved glycosylation of secondary alcohols bearing long alkyl chains with a terminal alkene which was subsequently utilized for late stage cross metathesis or oxidations (Jeong et al., 2005; Butcher et al., 2009; Martin et al., 2009; Noguez et al., 2012; Srinivasan et al., 2012; Hollister et al., 2013). Since we intended to study the binding affinity of the natural ascarosides and their intact fatty acid moieties independently, we synthesized the intact fatty acid side moieties **9** and **11** and coupled them directly to protected ascarylose **8** during the penultimate step of the synthetic sequence. This strategy provided rapid access to ascarosides **1** and **2** along with fatty acid derivatives **3-5** for screening. Lewis acid-mediated glycosylation with BF_3_•Et_2_O of fatty acid **9** (see **Supplementary Material** for synthesis details) and commercially available acid **11** (Jeong et al., 2005) proceeded as expected uneventfully and provided protected ascarosides **10** and **12** in 68 and 66% yield, respectively. Subsequent global deprotection with lithium hydroxide gave ascr#3 (**1**) and oscr#10 (**2**), Figure 2.

### *Na*-ASP-2 Binds Cholesterol and Palmitic Acid

**Figure 3:**
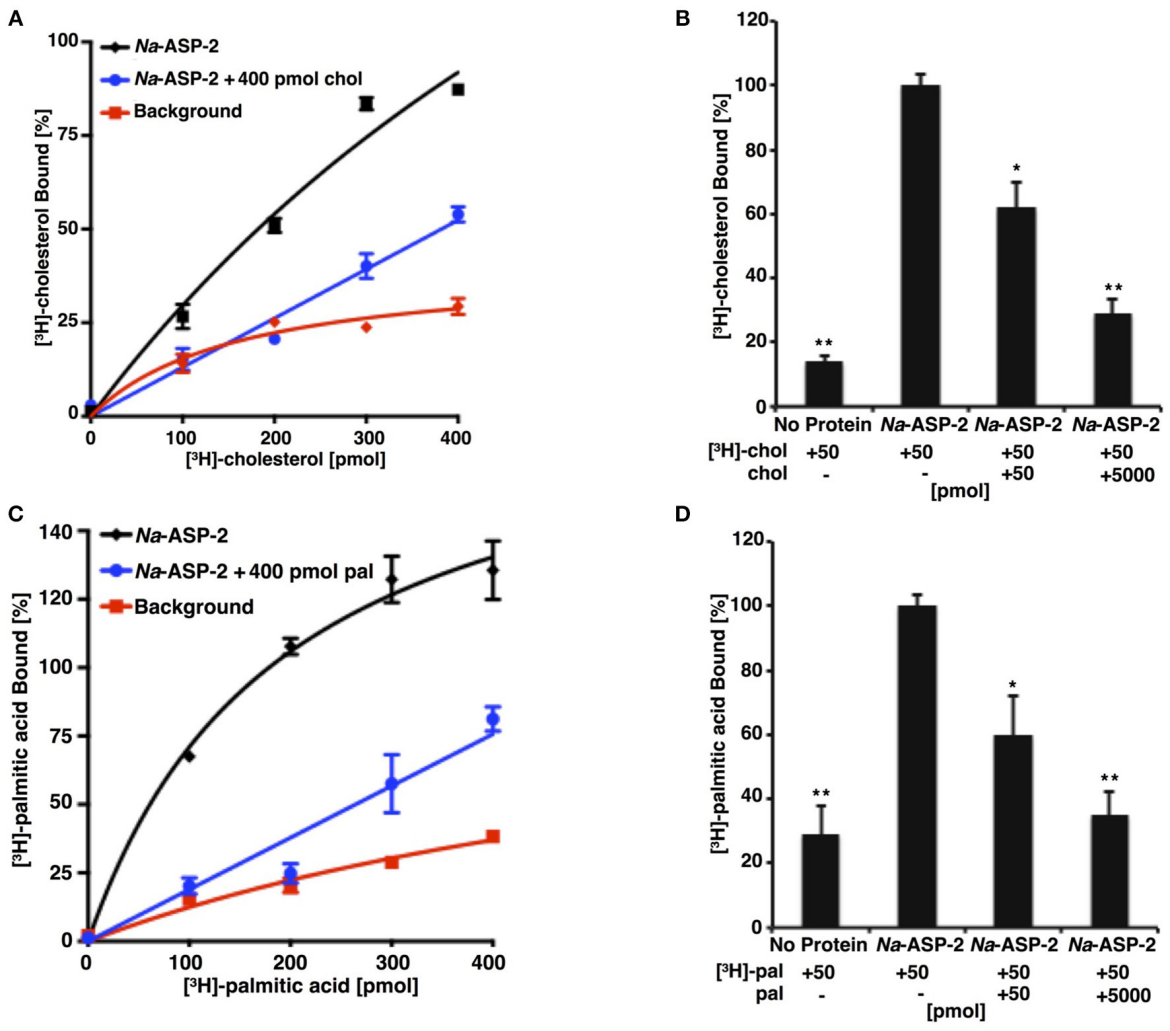
*Na*-ASP-2 binds both cholesterol and free palmitic acid. **(A)** Ligand binding of [^3^H]-cholesterol to *Na*-ASP-2. Purified *Na*-ASP-2 (100 pmol) was incubated with increasing concentrations of [^3^H]-cholesterol (100–400 pmol), in absence and presence of 400 pmol of unlabeled cholesterol (chol). The protein was separated from the unbound ligand by adsorption to an anion-exchange matrix and the protein-bound radioligand was quantified by scintillation counting. The background curve shows values obtained in the absence of added protein. Data represent mean ± SD of 3 independent experiments. **(B)** Competitive binding of unlabeled cholesterol (50 or 5,000 pmol) to *Na*-ASP-2. Binding of [^3^H]-cholesterol (50 pmol) to *Na*-ASP-2 (100 pmol) was assessed in the presence of the indicated concentrations of unlabeled cholesterol (chol). Each data point is the average of duplicate assays and represents the amount of [^3^H]-cholesterol bound relative to a control containing no unlabeled cholesterol. **(C)** Ligand binding of [^3^H]-palmitic acid to *Na*-ASP-2. Purified *Na*-ASP-2 (100 pmol) was incubated with increasing concentrations of [^3^H]-palmitic acid (100–400 pmol), in absence and presence of 400 pmol of unlabeled palmitic acid (pal). **(D)** Competitive binding of unlabeled palmitic acid (50 or 5,000 pmol) to *Na*-ASP-2. Binding of [^3^H]-palmitic acid (50 pmol) to *Na*-ASP-2 (100 pmol) was assessed in the presence of the indicated concentration of unlabeled palmitic acid (pal). Each data point is the average of duplicate assays and represents the amount of [^3^H]-palmitic acid bound relative to a control containing only labeled palmitic acid. Data represent mean ± SD of three independent experiments. Asterisks denote statistical significance relative to the control containing only the radiolabeled ligand and either purified *Na*-ASP-2 or Pry1. (**p < 0.001; *p < 0.01).

*Na*-ASP-2 has distinct sterol and palmitate binding cavities and can transport sterol *in vivo* (Darwiche et al., 2018). The *in vitro* cholesterol-binding activity of *Na*-ASP-2 was examined using increasing concentrations of radiolabeled [^3^H]-cholesterol and a constant concentration of purified protein, Figure 3A. Addition of equimolar or excess concentration of unlabeled cholesterol reduced binding of the radioligand, indicating that binding is specific, Figures 3A,B. *Na*-ASP-2 displayed saturable binding of cholesterol with an apparent dissociation constant *K*_*d*_ of 2.1 μM. *Na*-ASP-2 has similar cholesterol binding affinity as reported for other SCP/TAPS family members from yeast, *Saccharomyces cerevisiae* (Pry1, 1.9 μM), *Brugia malayi* (*Bm*-VAL-1, 0.9 μM), *Heligmosomoides polygyrus* (*Hp*-VAL-4, 1.53 μM), and *Schistosoma mansoni* (*Sm*-VAL-4, 2.4 μM) (Kelleher et al., 2014; Darwiche et al., 2016, 2018; Asojo et al., 2018).

Tablysin-15, a horsefly SCP/TAPS protein was shown to bind fatty acids via a hydrophobic pocket formed between two central helices (Ma et al., 2011). This hydrophobic pocket is observed in other SCP/TAPS proteins and we previously confirmed the ability of these proteins to bind palmitic acid *in vitro* (Kelleher et al., 2014; Darwiche et al., 2016, 2018; Asojo et al., 2018). To examine whether *Na*-ASP-2 can bind palmitic acid, we carried out direct binding studies using [^3^H]-palmitic acid as radiolabeled ligand, Figure 3C. For competition binding assays, binding of *Na*-ASP-2 to palmitic acid was reduced in the presence of unlabeled palmitic acid, indicating that binding is specific, Figure 3D. Based on the radioligand binding assay, *Na*-ASP-2 showed a saturable binding for palmitic acid with an apparent *K*_*d*_ of 95 μM, which is of the same magnitude as previously measured for the SCP/TAPS family members from yeast (Pry1, *K*_*d*_ = 112 μM), *Brugia malayi* (*Bm*-VAL-1, *K*_*d*_ = 83 μM), and comparable to tablysin-15 (*K*_*d*_ = 94 μM) (Kelleher et al., 2014; Darwiche et al., 2016, 2018; Asojo et al., 2018). Taken together our results indicate that *Na*-ASP-2 binds both cholesterol and palmitic acid *in vitro*. It is important to point out that the parasite proteins used for the competition assay do not have a His-tag and was 99+% pure protein that was previously used for crystallization studies. Tablysin-15 has a His-tag and was produced in *E. coli*. Similar *K*_*d*_ was measured for competition assays for Pry-1 using both hexa-histidine tagged or untagged protein and with protein produced from *E. coli* or *P. pastoris* and our previous studies indicate that the presence of the His-tag did not affect the ability to bind fatty acids and sterols (Darwiche et al., 2016, 2017a).

### Fatty Acids and Ascarosides Bind Selectively to the Palmitate-Binding Cavity

**Figure 4:**
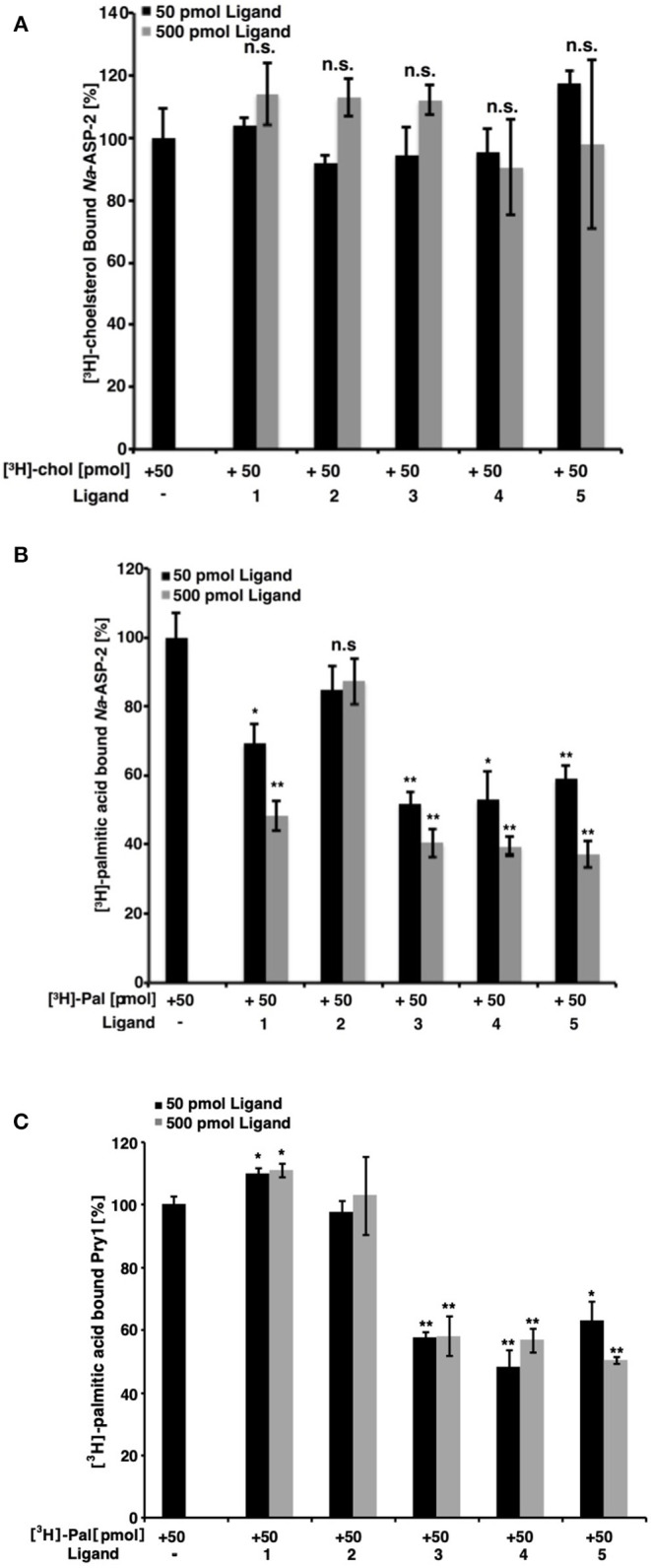
Binding of ligands to *Na*-ASP-2 and Pry1. **(A)** Free fatty acids and ascarosides fail to compete with [^3^H]-cholesterol for binding to *Na*-ASP-2. Binding of [^3^H]-cholesterol (50 pmol) to *Na*-ASP-2 (100 pmol) was assessed in the presence of (50 pmol and 500 pmol) of unlabeled ascarosides or fatty acid moieties (**1-5**) **(B)** Fatty acids moieties and ascr#3 compete with [^3^H]-palmitic acid for binding to *Na*-ASP-2. **(C)** Only fatty acids moieties compete [^3^H]-palmitic acid for binding to Pry1. Competitive binding was tested with either 50 or 500 pmol of the unlabeled ligands and 50 pmol of [^3^H]-palmitic acid for binding to 100 pmol purified *Na*-ASP-2 or Pry1. The ascarosides tested are (**1)** (ascr#3) and (**2)** (oscr#10) while the fatty acids are **3** [(*R*)-8-hydroxynonanoic acid], **4** [(*R, E*)-8-hydroxynon-2-enoic acid], and **5** (9-hydroxynonanoic acid). Data represent mean ± SD of 3 independent experiments. Asterisks denote statistical significance relative to the control containing only the radiolabeled ligand and either purified *Na*-ASP-2 or Pry1. (**p < 0.001; *p < 0.01). n.s., not significant.

Having confirmed the ability of *Na-*ASP-2 to bind cholesterol, we carried out competitive binding studies of ascarosides and their fatty acid moieties against radiolabeled cholesterol. At a concentration of 50 pmol, the typical concentration for our cholesterol binding assay, neither ascarosides [ascr#3 (**1**) and oscr#10 (**2**)] nor fatty acids (**3-5**) competed with the radiolabelled [^3^H]-cholesterol (50 pmol) for binding to *Na*-ASP-2, Figure 4A. We also tested if the ascarosides or their fatty acid moieties bind to the fatty acid binding cavity. Our studies showed that the binding of [^3^H]-palmitic acid by *Na*-ASP-2 was competed by the ascaroside, ascr#3 (**1**) and by all the fatty acid moieties **3**-**5** tested with the same order of magnitude, but not by the ascaroside, oscr#10 (**2**), Figure 4B. We tested the ability of Pry1, a SCP/TAPS protein from *S. cerevisiae*, an organism that does not contain ascarosides, to bind to the same ligands. Our analysis revealed that while the fatty acids (**3-5**) competed for palmitic acid binding to Pry1, neither ascr#3 (**1**) nor oscr#10 (**2**) bound to Pry1, Figure 4C. Furthermore, addition of excess ligands [fatty acids (**3-5**)] competed with radioligand binding while binding of [^3^H]-palmitic acid to Pry1 could not be competed for by the addition of excess unlabeled ascr#3 (**1**) or oscr#10 (**2**), Figure 4C. These competition studies reveal that *Na*-ASP-2 binds ascr#3 (**1**) through its fatty-acid binding pocket.

**Figure 5:**
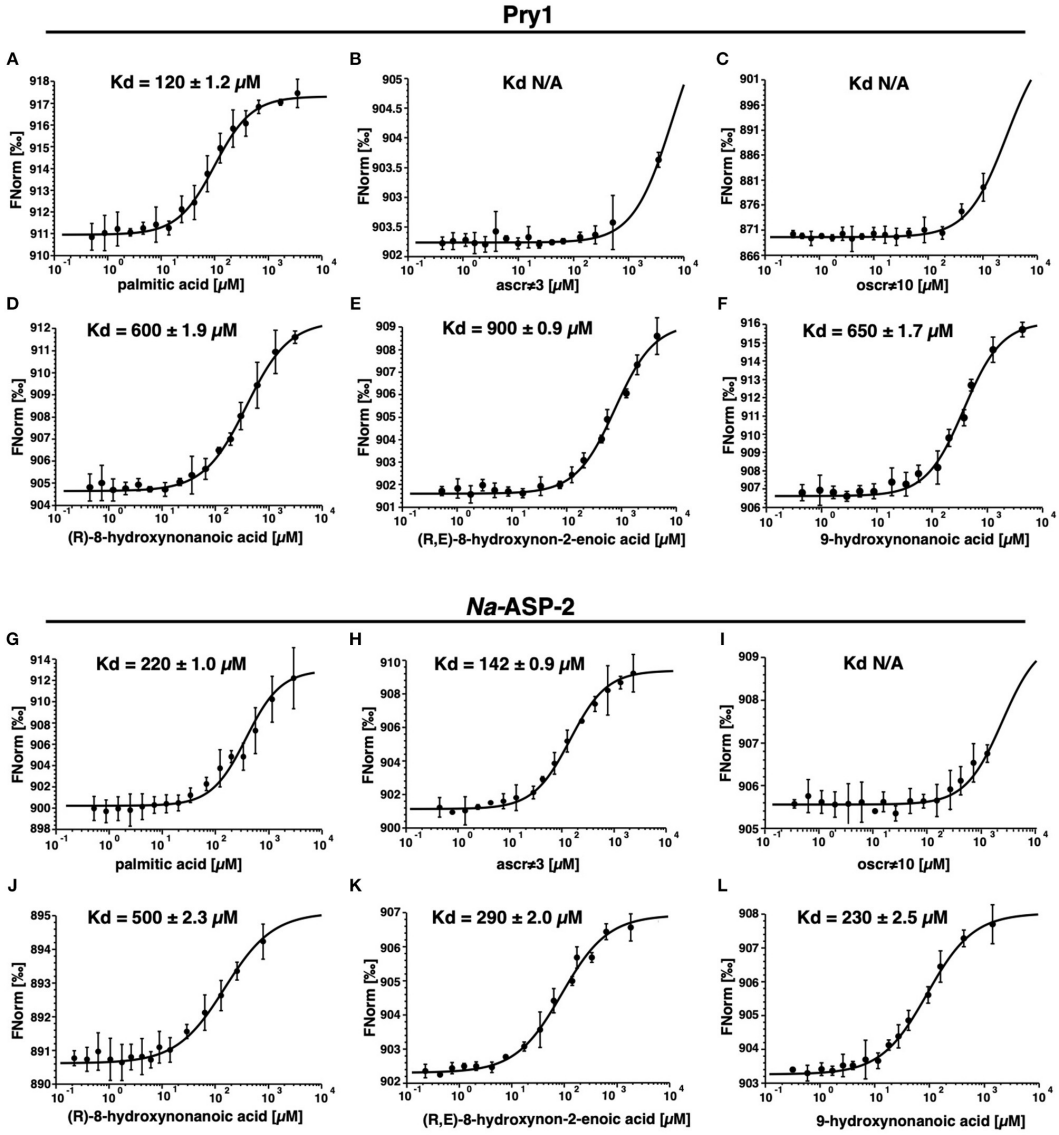
*Na*-ASP-2 selectively binds ascr#3 but not oscr#10. Binding of ascarosides and their fatty acid moieties by Pry1 and *Na*-ASP-2 as measured by microscale thermophoresis. **(A,G)** Palmitic acid; **(B,H)** ascr#3; **(C,I)** oscr#10; **(D,J)** (*R*)-8-hydroxynonanoic acid; **(E,K)** (*R, E*)-8-hydroxynon-2-enoic acid; **(F,L)** 9-hydroxynonanoic acid. Pry1 binds palmitic acid and free hydroxylated nanonoic acids with similar affinities but binds neither the ascarosides ascr#3 and oscr#10. Na-ASP-2 binds palmitic acid, ascr#3 and free hydroxylated nanonoic acids with similar affinities but not oscr#10. The *K*_*d*_ values are indicated in each figure with N/A (not applicable) where there is no binding. Data represent mean ± SD of three independent experiments.

We independently validated the direct binding of ligands to Pry1 and *Na*-ASP-2 by microscale thermophoresis and determined binding constants, Figure 5. Our analyses reveal that Pry1 does not bind ascr#3 or oscr#10, but it binds all the fatty acids tested including the moieties of both ascr#3 and oscr#10. *Na*-ASP-2, binds ascr#3 with a *K*_*d*_ of 142 μM but does not bind oscr#10. The *K*_*d*_ is in the same order of magnitude as the binding of palmitic acid and is consistent with the results obtained by the ligand competition assays (Kelleher et al., 2014; Darwiche et al., 2016, 2017a,b, 2018; Asojo et al., 2018).

## Discussion

**Figure 6:**
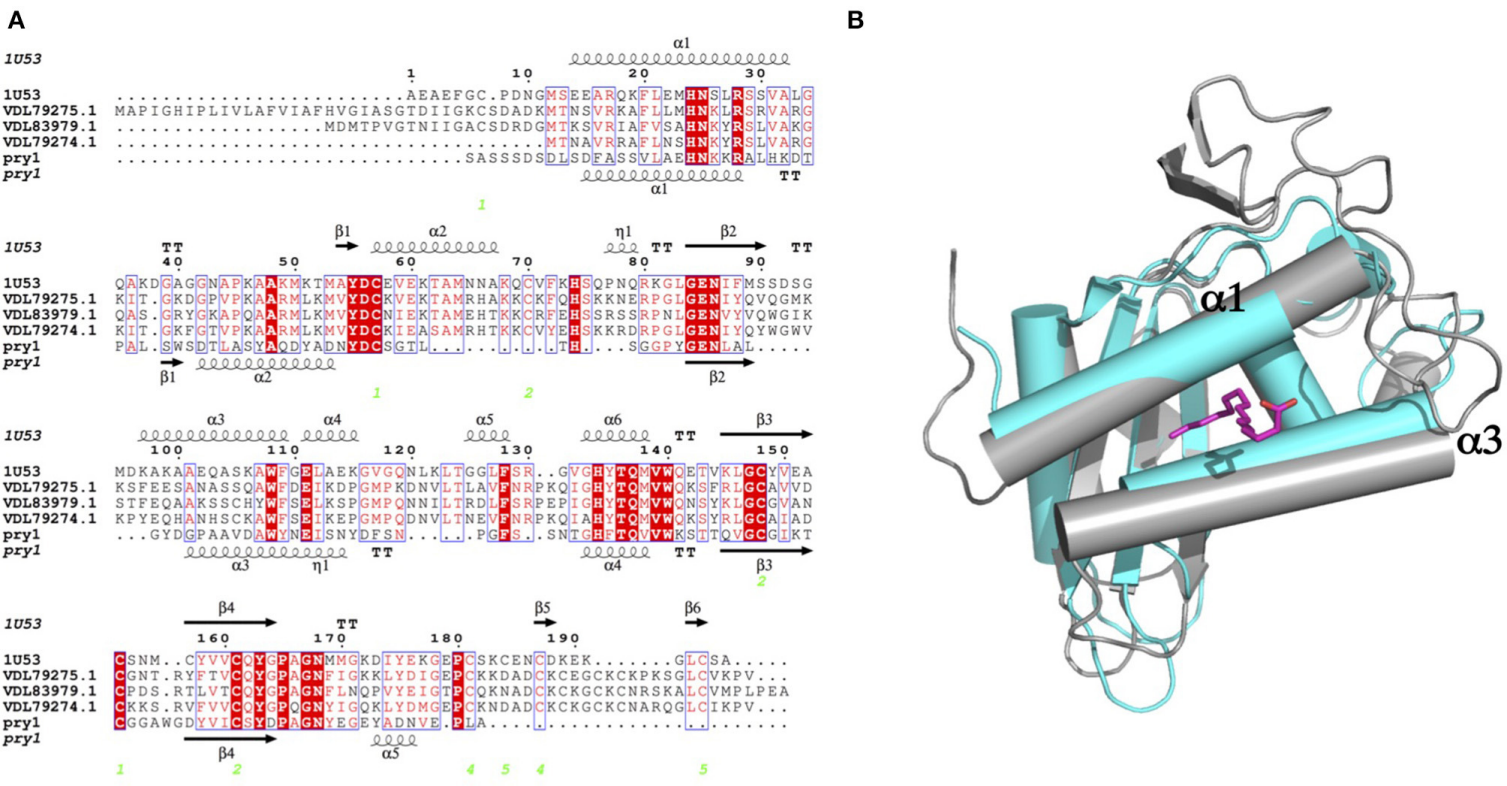
Comparison of fatty acid binding cavities of *Na*-ASP-2 and Pry1. **(A)** Structure based alignment of *Na*-ASP-2, Pry1, and three *N. brasilensis* SCP/TAPs proteins (genbank codes VDL79275.1; VDL83979.1; and VDL79274.1). The sequences are aligned with ClustalW Omega and the secondary structural features are illustrated with the coordinates of *Hp*-VAL-4 and Pry1 using ESPript (Gouet et al., 2003). The alpha helices (alpha 1 and alpha 3) that form the palmitate-binding cavity have similar lengths for *Na*-ASP-2 and the *N. brasilensis* proteins whereas Pry1 has shorter helices. The secondary structure elements shown are alpha helices (α), 3_10_-helices (h), beta strands (β), and beta turns (TT). Identical residues are shown in solid red, and conserved residues are in red. The locations of the cysteine residues involved in disulfide bonds are numbered in green. **(B)** Both of the helices (α1 and α3) forming the palmitic acid binding cavity of Pry1 (cyan) are shorter than those from *Na*-ASP-2 (gray). Also shown in magenta is the stick structure of palmitate superposed from the X-ray structure of the complex of tablysin-15 with palmitate (Ma et al., 2011).

We present here efficient methods to synthesize ascarosides and their fatty acid moieties. We also present data revealing that the fatty acid moieties of ascarosides compete for binding to the palmitate-binding cavities of both Pry1 and *Na*-ASP-2 but do not bind to the sterol binding cavity. The micromolar binding affinity of ascr#3 and free fatty acids are comparable to that observed for palmitic acid binding by other SCP/TAPS proteins (Kelleher et al., 2014; Darwiche et al., 2016, 2017a,b, 2018; Asojo et al., 2018). While it is unclear if ascr#3 binding is physiologically relevant, the finding that ascr#3 binds *Na*-ASP-2 is interesting considering that a high relative abundance of ascr#3 was detected in E-S products from both the infective juvenile and adult stages of rat hookworm (*Nippostrongylus brasilensis)* by HPLC-MS (Choe et al., 2012). It is plausible that ascr#3 is present in human hookworms since there appears to be a conservation of ascarosides production in families of nematodes (Choe et al., 2012). A blast search of the *Na*-ASP-2 sequence against the *N. brasilensis* proteins reveals several SCP/TAPs proteins, which share over 45% sequence similarity with *Na*-ASP-2. Even more remarkable, the residues and predicted structures of the helical regions notably residues corresponding to (α1 and α3) that form the fatty acid-binding cavity are partially conserved, Figure 6A. This structural similarity suggests that these proteins likely behave similarly to *Na*-ASP-2 as we observed previously for the orthologs from *B. malayi* and *H. polygyrus* (Asojo et al., 2018; Darwiche et al., 2018). Additionally, we observed that the incorporation of the ascarylose sugar abrogated the ability of these fatty acids to bind to Pry1. A comparison of the helices bordering the palmitic acid binding cavities of Pry1 and *Na*-ASP-2 reveals that Pry1 has shorter helices than *Na*-ASP-2, which results in a smaller hydrophobic binding pocket in Pry1 compared to *Na*-ASP-2, Figure 6B. This smaller size may explain the failure of Pry1 to accommodate ascarosides as opposed to free fatty acids. The inability of *Na*-ASP-2 to bind oscr#10 cannot be explained by the size difference of the cavities and suggests a new hypothesis that we plan to test in future; that ascarosides binding may be specific for certain SCP/TAPS proteins, indicating a possible functional relationship between ascarosides and parasite SCP/TAPS proteins.

The ability of *Na*-ASP-2 to bind cholesterol is intriguing given the evidence that hookworm and other parasite infections induce significant changes in lipid profile in patients suggesting that there may be some factors and proteins that help the parasite consume cholesterol (Bansal et al., 2005). As small soluble parasite proteins that can bind sterols and lipids, *Na*-ASP-2 and other parasite ASPs may play multiple roles in important processes that occur at the different life-stages during which they are produced. Hookworm infective larvae penetrate the skin in response to lipids during the transition from free-living to infectious state (Haas et al., 2005a,b). These free fatty acids were chemotactic stimuli for the skin penetration by hookworm larvae (Haas et al., 2005a,b). Additionally, the infective larvae of *N. americanus*, like other parasites, synthesize eicosanoids, which may stimulate inflammation and be important for immunomodulation and immune evasion (Salafsky and Siddiqui, 1990; Belley and Chadee, 1995). Previous studies have shown that eicosanoids bind to the fatty-acid binding cavity, and reported structures of tablysin-15 with either palmitate or an eicosanoid reveal similar binding in the same cavity (Ma et al., 2011). It is also possible that by binding to ascarosides and other small molecules, *Na*-ASP-2 may have roles in immune evasion or some other signaling cascade by infective *N. americanus* larvae. *Na*-ASP-2 is immunomodulatory and recruits neutrophil both *in vivo* and *in vitro* (Bower et al., 2008). Interestingly, *Na*-ASP-2 induced neutrophil recruitment appeared to be a mechanism of immune suppression by hookworm parasites (Tribolet et al., 2015). Similarly, adult hookworms secrete many proteins that have a potential immunomodulatory function, and among these are many ASPs. These proteins function by inhibiting the inflammatory reaction, promoting effector cells apoptosis, or skewing immune responses to help hookworms survive inside the host (Loukas and Prociv, 2001). Clarifying how some of these proteins bind host and parasite small molecules and proteins offers insights into host-parasite interactions. Understanding how *Na*-ASP-2 interacts with host and parasite molecules is also important because recombinant *Na*-ASP-2 remains an interesting hookworm vaccine candidate, especially when modified to decrease its allergenicity or used as a pediatric vaccine before the development of anti-hookworm IgE (Zhan et al., 2012).

## Conclusions

In summary, our results reveal that the fatty acid moieties of ascarosides, ascr#3 and oscr#10 bind *Na*-ASP-2 and Pry1, with the latter SCP/TAPS protein from *Saccharomyces cerevisiae* serving as a control. As shown by the palmitic acid competition assay, binding is through the fatty acid binding cavity. Additionally, ascr#3 an ascaroside that is present in mammalian hookworm E-S products binds to *Na*-ASP-2. Oscr#10 which is not found in hookworm E-S products does not bind to *Na*-ASP-2. Neither ascr#3 nor oscr#10 bind Pry1. Future studies will identify how ascarosides precisely interact with parasite SCP/TAPS protein and determine the physiological relevance of the fatty acid-binding cavity of *Na*-ASP-2.

## Data Availability Statement

The raw data supporting the conclusions of this article will be made available by the authors, without undue reservation. Publicly available datasets were analyzed in this study. This data can be found here: Genbank, VDL79275.1; VDL83979.1; and VDL79274.1.

## Author Contributions

OA, OEA, RS, RD, KH, and DR: designed the studies. OEA, RD, and NT: conducted experiments. All authors contributed expertise and to the final manuscript, in doing so, all authors agree to be accountable for the content of the work.

## Conflict of Interest

The authors declare that the research was conducted in the absence of any commercial or financial relationships that could be construed as a potential conflict of interest.
